# Supplementary material for: WGCNA Identifies Translational and Proteasome-Ubiquitin Dysfunction in Rett Syndrome
Source: Int J Mol Sci. 2021 Sep 15;22(18):9954. doi: 10.3390/ijms22189954 (PMC8465861; doi:10.3390/ijms22189954)
Supplement: Supplementary file 1 [file ijms-22-09954-s001.zip › ijms-1297897-supplementary.pdf]

Supplementary Material

Supplemtary Table S1 Source of datasets: GSE access number, sample IDs, cell types, status (diseased, isogenic, healthy control), gender (F, female), MeCP2 mutations and location in the loci (Methyl binding domain (MBD), C-terminus, Transcriptional repressor domain (TRD) and WW domain.), and phenotype of patient/control (data source of Biobank Coriell Institute is indicated)

	Sample ID	Cell type	Status	Gender	Mutation (protein and nucleotide change)	Location	Phenotype
GSE51607<br>Tanaka et al.	RTT1.iPS.13w	iPSCs	Isogenic	F	p.T158M<br>c. 473C>T	MBD	Isogenic control<br>GM17880-Coriell Institute
	RTT5.iPS.31w	iPSCs	Isogenic	F	p.X487W<br>c.1461A>G	C-terminus	Isogenic control<br>GM17567-Coriell Institute
	RTT4.iPS.24w	iPSCs	Isogenic	F	p.R306C<br>c.916C>T	TRD	Isogenic control<br>GM11270-Coriell Institute
	RTT5.iPS.42m	iPSCs	Disease	F	p.X487W<br>c.1461A>G	C-terminus	Clinically affected; onset between 15-20 months of age; seizures began at age 3; never walked independently; began to develop repetitive hand movements at 28 months; no hand use; small feet; language regression at 18 months; some sleep problems; nonverbal; significantly abnormal EEG; swallowing difficulties, reflux, and breathing problems; teeth grinding; decelerating head circumference; growth retardation; seizures; donor subject has a missense mutation (A>G) at nucleotide 1461 (1461A>G) in the gene encoding methyl-CpG binding protein 2 (MECP2), resulting in a substitution of a tryptophan for a stop codon at codon 487 [TER487TRP (X487W)]. 5 years old at time of sampling.<br>GM17567-Coriell Institute
	RTT5.iPS.34m	iPSCs	Disease	F	p.X487W<br>c.1461A>G	C-terminus	As above
	RTT3.iPS.16m	iPSCs	Disease	F	p.E235fs	TRD	Growth and developmental delay; can walk only with assistance; nonverbal; no hand use; constant repetitive hand motions; no seizures, but significantly abnormal EEG; teeth grinding; some sleep difficulties; eating problems with minor reflux; breath holding and hyperventilation; small feet; some tremor; this culture had a lifespan of 56 population doublings (PDLs); the donor subjects carries a C>T transition at nucleotide 473 (ACG>ATG) in exon 3 of the gene encoding methyl-CpG binding protein 2 (MECP2) resulting in the substitution of threonine 158 by methionine [Thr158Met (T158M)] in the methyl-binding domain; see GM16271 (lymphocyte) 5 yrs old at time of biopsy.<br>GM07982-Coriell Institute
	RTT4.iPS.16m	iPSCs	Disease	F	p.R306C<br>c.916C>T	TRD	Clinically affected; classical symptoms; normal lysosomal enzymes; 46,XX in PBL; donor carries a missense mutation, 916C>T [Arg306Cys (R306C)], in the gene encoding methyl-CpG binding protein 2 (MECP2). 8 years old at time of sampling.<br>GM11270-Coriell Institute
GSE107399<br>Ohashi et al.	Control	iPSCs	WT	F	-	-	-
	Control 1<br>(rep 1 and 2)	iPSC	Isogenic	F	p.X487W<br>c.1461A>G	C-terminal	-
	Control 2<br>(rep 1and 2)	iPSCs	Isogenic	F	p.V247X<br>c.705delG	TRD	-

	RTT_1 (rep 1 and 2)	iPSCs	Disease	F	p.X487W c.1461A>G	C-terminus	Clinically affected; onset between 15-20 months of age; seizures began at age 3; never walked independently; began to develop repetitive hand movements at 28 months; no hand use; small feet; language regression at 18 months; some sleep problems; nonverbal; significantly abnormal EEG; swallowing difficulties, reflux, and breathing problems; teeth grinding; decelerating head circumference; growth retardation; seizures; donor subject has a missense mutation (A>G) at nucleotide 1461 (1461A>G) in the gene encoding methyl-CpG binding protein 2 (MECP2), resulting in a substitution of a tryptophan for a stop codon at codon 487 [TER487TRP (X487W)]. 5 years old at time of sampling. GM17566-Coriell Institute
	RTT_2 (rep 1 and 2)	iPSCs	Disease	F	p.V247X c.705delG	TRD	Clinically affected; microcephaly; scoliosis diagnosed at age 12; severe kyphoscoliosis at age 25; early milestones were slow; started losing skills at age 2; currently severely retarded; behavioral phenotype includes hand wringing that began at age 2 and became more intense; no sleep problems; no self-injurious behavior; abnormal EEG; CT scan at age 25 showed evidence of atrophy; donor subject carries a frameshift mutation, 705delG, in the gene encoding methyl-CpG binding protein 2 (MECP2); lymphoblast. GM0798-Coriell Institute
GSE21307 Marchetto et al.	Cnl15Rep1	iPSCs	disease	F	c.1155del32	WW	Clinically affected; classical symptoms; normal lysosomal enzymes; 46,XX in PBL; donor subject carries a frameshift mutation, 1155del32, in the gene encoding methyl-CpG binding protein 2 (MECP2). GM11272-Coriell Institute
	Cln15Rep2	iPSCs	disease	F	p.L386Rfs c.1155del32	WW	As above
	Cln15Rep3	iPSCs	disease	F	p.L386Rfs c.1155del32	WW	As above
	Cln18Rep1	iPSCs	disease	F	p.L386Rfs c.1155del32	WW	As above
	Cln18Rep2	iPSCs	disease	F	p.L386Rfs c.1155del32	WW	As above
	Cln18Rep3	iPSCs	disease	F	p.L386Rfs c.1155del32	WW	As above
	WT Cln1Rep1	iPSCs	Paediatric control	F	-	-	The culture was initiated on 1/22/87 from explants of minced gum tissue removed ante-mortem. The cell morphology is fibroblast-like. The karyotype is 46,XX; normal diploid female. AG09319 Fibroblast from Gingival Gum-Coriell Institute
	WT Cln1Rep1	iPSCs	Paediatric control	F	-	-	As above
	WT Cln1Rep3	iPSCs	Paediatric control	F	-	-	As above
	WT Cln2Rep1	iPSCs	Paediatric control	F	-	-	As above

	WT Cln2Rep2	iPSCs	Paediatric control	F	-	-	As above
	WT Cln2Rep3	iPSCs	Paediatric control	F	-	-	As above

Supplementary Table S2. *Pathway enrichment results using KEGG for significantly correlated modules*

Dataset	Module Color	Enrichment	Term ID	Term description	Observed gene count	Background gene count	Strength	False discovery rate
MT	black	No significant enrichment detected -			-			
	floralwhite		hsa05016	Huntington's disease	15	193	0.62	0.0017
			hsa00190	Oxidative phosphorylation	11	131	0.66	0.0075
			hsa04714	Thermogenesis	13	228	0.49	0.0406
			hsa05012	Parkinson's disease	10	142	0.58	0.0406
	yellowgreen	No significant enrichment detected			-			
	mediumorchid	No significant enrichment detected			-			
	plum4	No significant enrichment detected			-			
	mistyrose	No significant enrichment detected			-			

	lavanderblush		hsa03008	Ribosome biogenesis in eukaryotes	22	76	0.53	0.002
			hsa00970	Aminoacyl-tRNA biosynthesis	15	66	0.47	0.003
			hsa04141	Protein processing in endoplasmic reticulum	32	161	0.35	0.0176
			hsa03050	Proteasome	12	46	0.58	0.023
TK	orange	No significant enrichment detected						

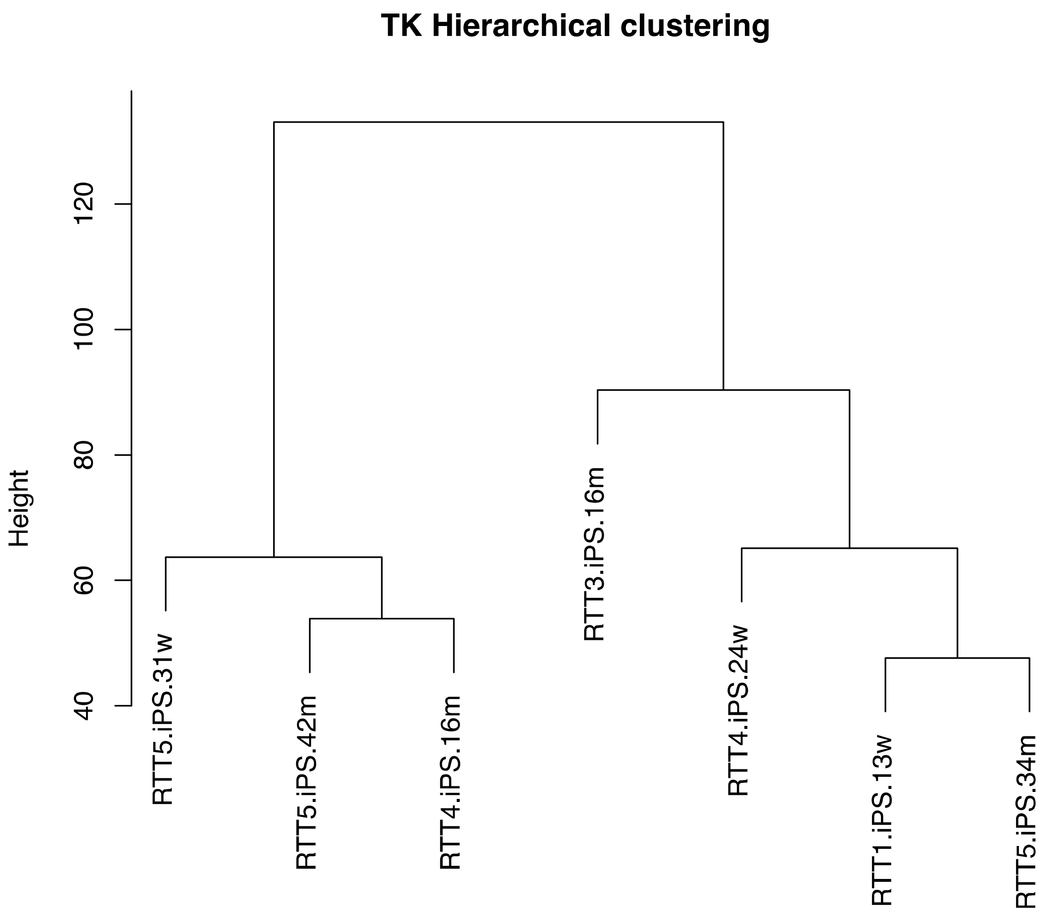
TK Hierarchical clustering
Height
40
60
80
100
120
RTT5.iPS.31w
RTT5.iPS.42m
RTT4.iPS.16m
RTT3.iPS.16m
RTT4.iPS.24w
RTT1.iPS.13w
RTT5.iPS.34m

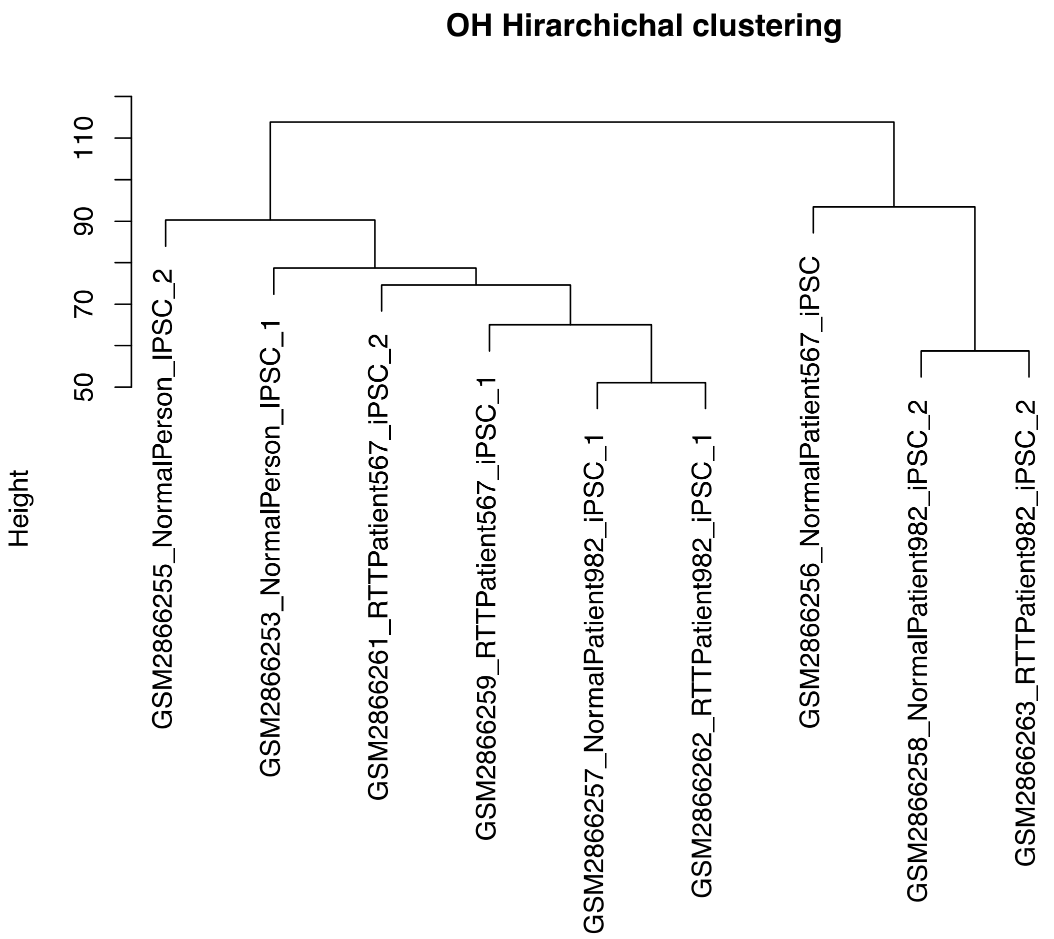
OH Hirarchichal clustering
Height
50
70
90
110
GSM2866255_NormalPerson_IPSC_2
GSM2866253_NormalPerson_IPSC_1
GSM2866261_RTTPatient567_iPSC_2
GSM2866259_RTTPatient567_iPSC_1
GSM2866257_NormalPatient982_iPSC_1
GSM2866262_RTTPatient982_iPSC_1
GSM2866256_NormalPatient567_iPSC
GSM2866258_NormalPatient982_iPSC_2
GSM2866263_RTTPatient982_iPSC_2

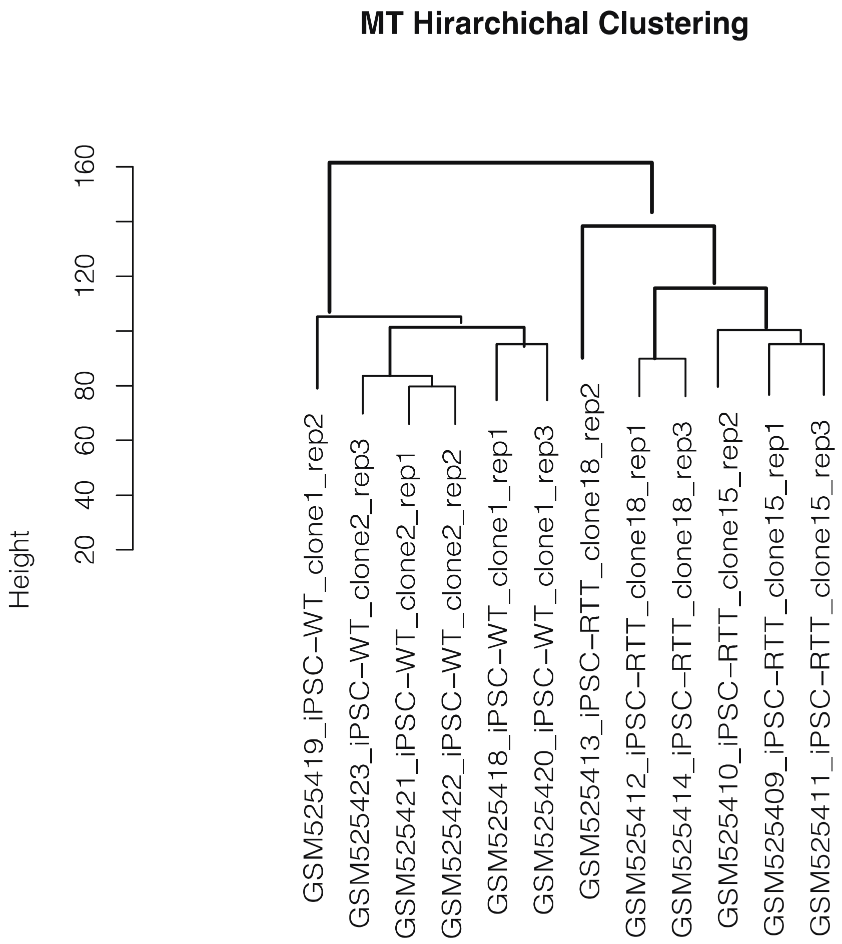

Supplementary Figure S1. *Hierarchical clustering of iPSCs samples used in each study.*
